# Autonomous Navigation System of Greenhouse Mobile Robot Based on 3D Lidar and 2D Lidar SLAM

**DOI:** 10.3389/fpls.2022.815218

**Published:** 2022-03-10

**Authors:** Saike Jiang, Shilin Wang, Zhongyi Yi, Meina Zhang, Xiaolan Lv

**Affiliations:** ^1^School of Agricultural Engineering, Jiangsu University, Zhenjiang, China; ^2^Institute of Agricultural Facilities and Equipment, Jiangsu Academy of Agricultural Science, Nanjing, China; ^3^Key Laboratory for Protected Agricultural Engineering in the Middle and Lower Reaches of Yangtze River, Ministry of Agricultural and Rural Affairs, Nanjing, China

**Keywords:** greenhouse, mobile robot, navigation, Lidar, SLAM

## Abstract

The application of mobile robots is an important link in the development of intelligent greenhouses. In view of the complex environment of a greenhouse, achieving precise positioning and navigation by robots has become the primary problem to be solved. Simultaneous localization and mapping (SLAM) technology is a hot spot in solving the positioning and navigation in an unknown indoor environment in recent years. Among them, the SLAM based on a two-dimensional (2D) Lidar can only collect the environmental information at the level of Lidar, while the SLAM based on a 3D Lidar demands a high computation cost; hence, it has higher requirements for the industrial computers. In this study, the robot navigation control system initially filtered the information of a 3D greenhouse environment collected by a 3D Lidar and fused the information into 2D information, and then, based on the robot odometers and inertial measurement unit information, the system has achieved a timely positioning and construction of the greenhouse environment by a robot using a 2D Lidar SLAM algorithm in Cartographer. This method not only ensures the accuracy of a greenhouse environmental map but also reduces the performance requirements on the industrial computer. In terms of path planning, the Dijkstra algorithm was used to plan the global navigation path of the robot while the Dynamic Window Approach (DWA) algorithm was used to plan the local navigation path of the robot. Through the positioning test, the average position deviation of the robot from the target positioning point is less than 8 cm with a standard deviation (SD) of less than 3 cm; the average course deviation is less than 3° with an SD of less than 1° at the moving speed of 0.4 m/s. The robot moves at the speed of 0.2, 0.4, and 0.6 m/s, respectively; the average lateral deviation between the actual movement path and the target movement path is less than 10 cm, and the SD is less than 6 cm; the average course deviation is <3°, and the SD is <1.5°. Both the positioning accuracy and the navigation accuracy of the robot can meet the requirements of mobile navigation and positioning in the greenhouse environment.

## Introduction

With the development of mechanization and automation, agriculture has undergone an accelerated upgrading toward information and intelligent agriculture in the world. Also, with the development of high technology and with the incremental labor cost, the application of robots in agriculture has become more and more extensive. Compared with the complex field environment, the greenhouse environment is relatively simple; however, in greenhouses where plants are densely distributed under high temperature and high humidity, and, sometimes, even toxic gases are emitted, there are some potential safety hazards in manual operation (Henten et al., 2013). Therefore, robots enjoy a large application market in picking, plant protection, inspection, and other aspects of greenhouses (Uyeh et al., 2019).

In terms of the autonomous navigation of robots, the navigation solutions based on Global Navigation Satellite System (GNSS) have been fully applied in the field operations environment (Pérez Ruiz and Upadhyaya, 2012). However, as there are many obstructions in greenhouses to cause the loss of satellite signals, the greenhouse environment is not suitable for mobile robots. Path planning and movement, which are safe, fast, and effective, have become the primary difficulties in the application of greenhouse mobile robots.

The guide rail navigation is a common navigation solution for greenhouse mobile robots. It realizes the mobile navigation in greenhouses by laying rails on the ground (Chiu et al., 2013; Hayashi et al., 2014; Lee et al., 2015). Considering the high cost of the rail laying and the occupation of the ground in greenhouses, some robots that use greenhouse pipes as motion guide rails have been developed, and these robots can move along the pipes or on the ground (Zhao et al., 2016; Arad et al., 2020). To further improve the safety and practicability of the robots on guide rails, Balaso et al. (2013) installed a distance sensor, a photoelectric sensor, and an ultrasonic sensor to assist the navigation of the designed multi-functional greenhouse robot. Although the guide rail navigation is simple in operation, the fixed rails greatly limit the movement path and the range of the robot. Magnetic navigation and ribbon navigation through the magnetic stripes and ribbons replace the rails in the guide rail navigation. Magnetic navigation realizes path tracking by detecting the electromagnetic signals installed on the ground (Pan et al., 2019), and the color band navigation uses visual sensors to detect the edge of the color band to achieve navigation (Min et al., 2014). Compared with the guide rail navigation, the installation cost of magnetic stripes and color bands is relatively low and does not occupy the greenhouse space; moreover, their laying and installation are simpler and more flexible. Nevertheless, they could not get rid of the movement restrictions by fixed routes.

Machine vision navigation uses monocular vision or stereo vision sensors to collect environmental information then extract the navigation paths or crop lines based on the Hough Transform (Hough, 1962), the least-Square Methods (Cui et al., 2015; Mao et al., 2019), and the binocular stereo vision algorithms (Zou et al., 2012). Wang et al. (2012) analyzed the distribution characteristics of each component of the road image between the tomato ridges in the Hue, Saturation and Intensity (HSI) color space and then proposed a greenhouse tomato path detection method between the ridges based on the least square method. The experimental results showed that the proposed method could accurately extract the edge information of the target sensitive area; there was a 91.67% accuracy rate of extracting the navigation path between the tomato ridges with different coverage. In view of the problems of poor recognition of visual navigation technology and vulnerability to illumination, Gao and Ming (2014) selected the H component in the HIS color space for subsequent image processing and introduced the K-means algorithm to cluster and to segment the image for the unique color characteristic information of greenhouse. Chen et al. (2021) proposed a Hough transform algorithm for the prediction point by using a new graying factor to segment cucumber plants and soil, and this proposed algorithm is used for prediction points to fit the navigation paths. This algorithm is 35.20 ms faster than the traditional Hough Transform. The robot uses the machine vision sensor, which is carried by itself to autonomously navigate, thus saving the cost of setting up the environment in the early stage. However, the navigation path of the robot needs to be fitted after extracting the greenhouse vegetation or the roadside information each time, so the path of the robot is subject to environmental constraints, which further limits the space for robot movements.

The positioning and navigation method, based on multi-source data fusion, is the current hotspot in the research of the navigation of greenhouse mobile robots. In this navigation environment, the robot can move freely within a greenhouse. In general, the navigation by fusing multi-source data can be divided into two types: one is to achieve precise positioning and navigation by arranging sensors in the environment with the assistance of an inertial measurement unit (IMU), an odometer, and other modules that are carried by the robot itself; the other is to achieve the positioning and navigation directly by the sensor that is carried by the robot itself. Widodo et al. (2012) applied the acoustic positioning system in the greenhouse for the first time. To reduce the time consumed in manual deployment and calibration, Widodo et al. (2013) subsequently designed a self-calibrating acoustic positioning system. Huang et al. (2020a) proposed a spread spectrum sound-based local positioning system for greenhouse robots, and Tsay et al. (2020) added a temperature compensation on this basis. In addition to a sound-based positioning system, Preter et al. (2018) designed a strawberry harvesting robot, which uses an ultra-wideband indoor positioning system, wheel encoders, and a gyroscope to achieve positioning and navigation in the greenhouse. The development of indoor navigation technologies, such as radio frequency identification (Choi et al., 2011; Ming, 2018), Bluetooth low energy (Spachos et al., 2021), and positioning by signal strength (Huang et al., 2020b), provide more options for greenhouse mobile robot navigation. However, for all the above navigation solutions, it is necessary to arrange base stations, tags, and other external sensors in advance in the greenhouse. Although the installation procedures are much simpler than the guide rail navigation, the magnetic navigation, and the color band navigation, the technology of positioning and navigation through the sensors, as carried by the robot itself, eliminates these extra steps. In an unknown environment, the robot uses the sensors it carries to achieve navigation. The first and most important thing is that the robot knows its location. The SLAM technology can help the robot build an environment map and estimate its posture well. According to the types of sensors, the SLAM technology can be divided into visual SLAM technology and Lidar SLAM technology. In comparison, since the visual sensor is susceptible to the influence of light intensity, the visual SLAM technology has poor mapping performance in the poor light environment; while the Lidar SLAM technology is not affected by light, with higher accuracy, less calculation, and more mature technology (Chan et al., 2009). The SLAM technology, based on two-dimensional (2D) Lidar, has achieved good results in the research of greenhouse mobile robot navigation (Juan et al., 2016; Obregón et al., 2019; Hou et al., 2020; Tiwari et al., 2020). However, the environment detected by 2D Lidar is only on the same horizontal plane as the installation position of the Lidar on the robot. More stringent requirements are needed for the installation of Lidar and the greenhouse environment. At the same time, the Lidar cannot detect the environmental information above and below itself. Therefore, it leaves a huge potential safety hazard in robot navigation. The SLAM technology, based on three-dimensional (3D) Lidar, can detect all the environmental information of the greenhouse, which enhances the safety of the robot when it moves, but it also increases the computational burden of the robot and puts forward higher requirements on the computational performance of the robot.

Based on the Robot Operating System (ROS), this study proposed a new positioning and navigation solution for greenhouse mobile robots by combining the SLAMs of both 3D Lidar and 2D Lidar. First, the 3D point cloud data, collected by multi-line Lidar, were filtered and were fused into 2D data. The 2D information after the fusion contained the location information of key points, within the motion range of the robot, to the maximum extent. Then, the 2D Lidar SLAM algorithm, based on the encoder information and IMU information, was used to build the environment map, and the optimal navigation path was further planned to achieve the positioning and navigation of the greenhouse mobile robot, which not only ensured the safety of the robot mapping navigation but also reduced the energy consumption in data calculation by the robot.

## Materials and Methods

### Hardware System Design

**Figure 1:**
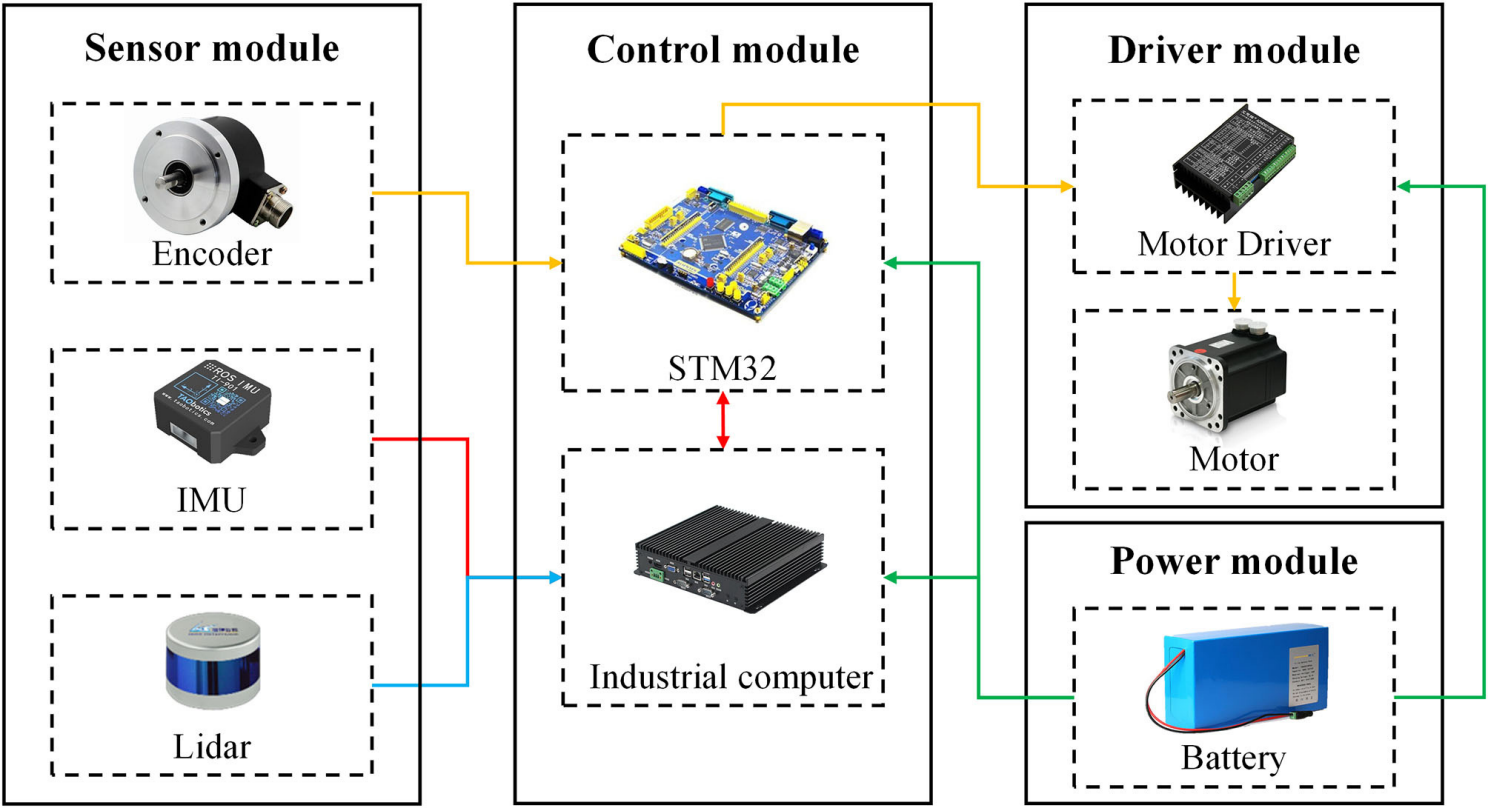
Hardware structure diagram of the robot navigation system.

The designed hardware system of the greenhouse mobile robot is mainly composed of the sensor module, the control module, the driver module, and the power module. The hardware system structure is shown in Figure 1.

#### Sensor Module

The sensor module of the robot was mainly composed of an encoder, an IMU, and a Lidar. The encoder is composed of a 1,024-line photoelectric incremental code disc, which collected the real-time speed information of the robot and sent feedback to the bottom controller of the robot. The inertial measurement unit has the 9-axis IMU (HFI-A9, HandsFree, Shenzhen, China), which includes a 3-axis gyroscope, a 3-axis accelerometer, and a 3-axis magnetometer. The internal integrated posture solver, with the assistance of the dynamic Kalman filter algorithm, can accurately output the real-time posture of the robot in a dynamic environment, thus providing accurate calculation data for the determination of the position of the robot in the greenhouse, such as Euler angles, quaternions, and the most commonly used roll/pitch/yaw direction data. The Lidar contains 16 pairs of Lidar transmitting and receiving modules (C16, Leishen Intelligent, Shenzhen, China). By adopting the time of fight measurement method, with a vertical resolution of 1.33°, the internal motor can be driven at a speed of 5 Hz (or 10 or 20 Hz) for 360° scanning. The 100M Ethernet UDP/IP communication protocol is used for data output and configuration.

#### Control Module

The STM32F103 embedded system board was adopted in the bottom controller of the robot. The core of the system board is a 32-bit high-performance ARM Cortex-M3 processor with a maximum operating frequency of 72 MHz. It has built-in high-speed memory, abundant enhanced I/O ports, and peripherals connected to two advanced peripheral buses (APBs). The power supply voltage is 2– to 3.6 V, and a series of power-saving modes can ensure the needs of the low-powered applications. The bottom controller is connected to the motor driver, the encoder, and the upper computer. According to the real-time speed information provided by the encoder, the STM32 can use the classic proportional-integral-derivative (PID) algorithm to control the motor rotation through the motor driver, to realize the precise movement of the robot. In addition, to enhance the safety of the robot during the movement, the bottom controller will limit the output of the driver and pull up the robot slowly when the temperature of the motor driver is higher than the protection temperature.

The top control of the robot was equipped with an industrial computer as the upper computer (EPC-P3086, Dongtintech, Shenzhen, China), and the Ubuntu18.04 operating System and ROS were installed respectively. The bottom control and the top control of the robot were connected through the control area network (CAN) bus protocol. The communication baud rate is 500K and the message format is MOTOROLA. Through the CAN bus interface, the PC can realize the control of the linear velocity and angular velocity of the mobile robot. Meanwhile, the PC will also receive real-time feedback of the motion state information of the robot.

#### Driver Module

A total of four 200-W DC brushless servo motors (SDGA-02C11AB24, Tode, Jiaxing, China) were installed at the front and rear of the robot, and a gearbox of 1:30 was equipped to provide sufficient power for the robot (60TDF-147050-L2-H, Tode, Jiaxing, China). The no-load maximum speed is 1.5 m/s. The driving form of the robot was four-wheel independent driving, using a four-wheel differential steering, which could realize a spot turn. In addition to the above functions, the power module of the robot also adopted the composite design of inflatable rubber wheels and independent suspension, which equipped the robot with strong ground clearance and ground adaptability. The robot climbing angle is up to 30°, and the minimum clearance from the ground is 135 mm, which can meet the flexible movement of the robot on different types of ground in the greenhouse.

#### Power Module

The power module of the robot adopted a 24-V ternary lithium battery (LS-DL24-30, Lishen Energy, Shenzhen, China). The battery voltage is about 29.2 V when it is fully charged with the capacity of 30 Ah. It has a built-in voltage regulator module and a power display module. Under normal circumstances, it can supply power continuously for 3–5 h. When the battery voltage is less than 22.5 V, the robot chassis will automatically alarm with a buzzer, and it will take about 3 h to fully recharge.

### Software System Design

**Figure 2:**
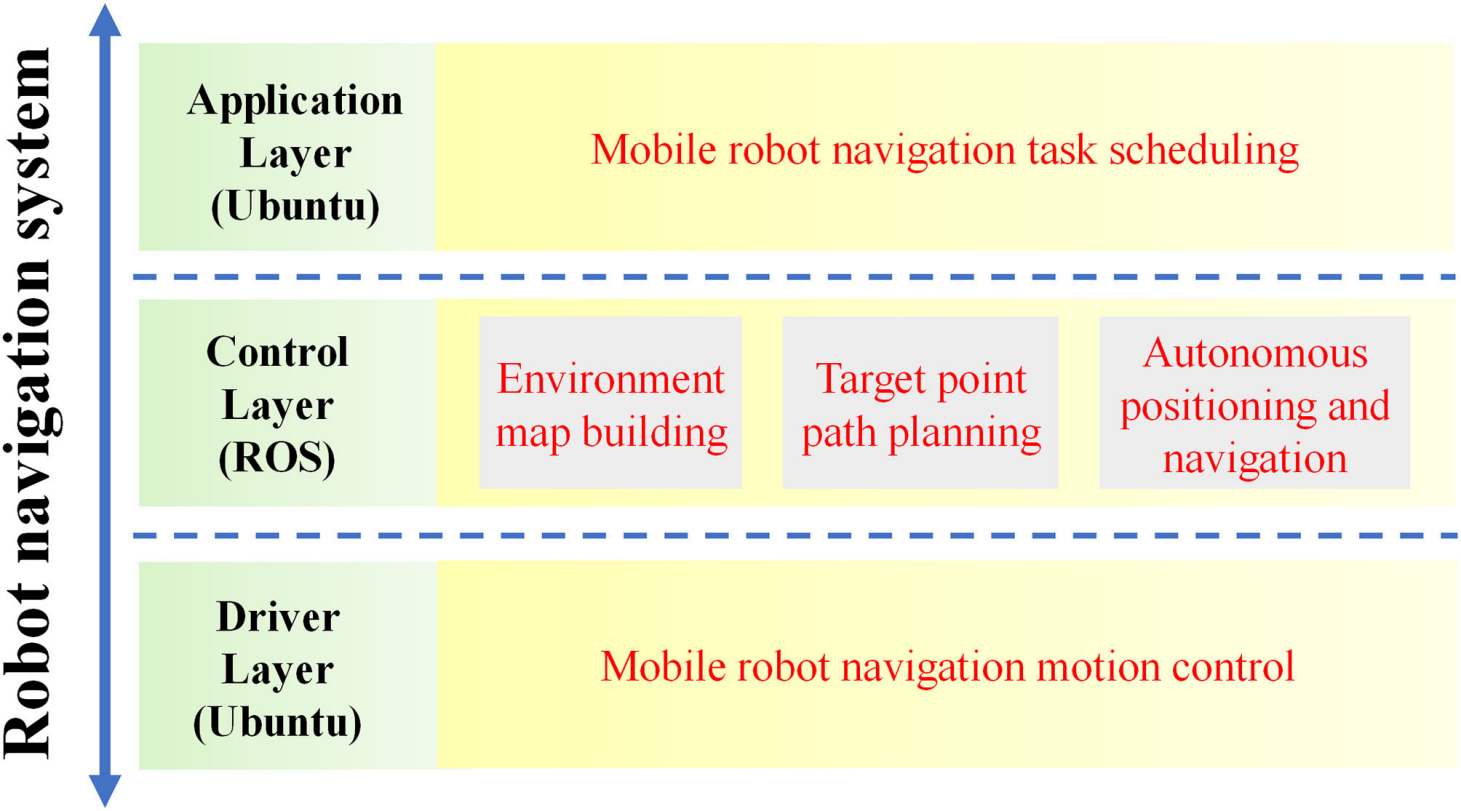
Software structure diagram of the robot.

The overall software system of the greenhouse mobile robot was designed based on Ubuntu 18.04, as shown in Figure 2. It included an application layer, a control layer, and a driver layer. The most important part was the control layer, which was developed based on ROS. It was responsible for the collection, fusion, and processing of information from robot sensors and then for completing the map construction, path planning, and autonomous positioning and navigation according to control instructions. The ROS has a distributed architecture that allows each functional module in the framework to be individually designed, compiled, and loosely coupled together at run time.

### Implementation Principles of Navigation Function

**Figure 3:**
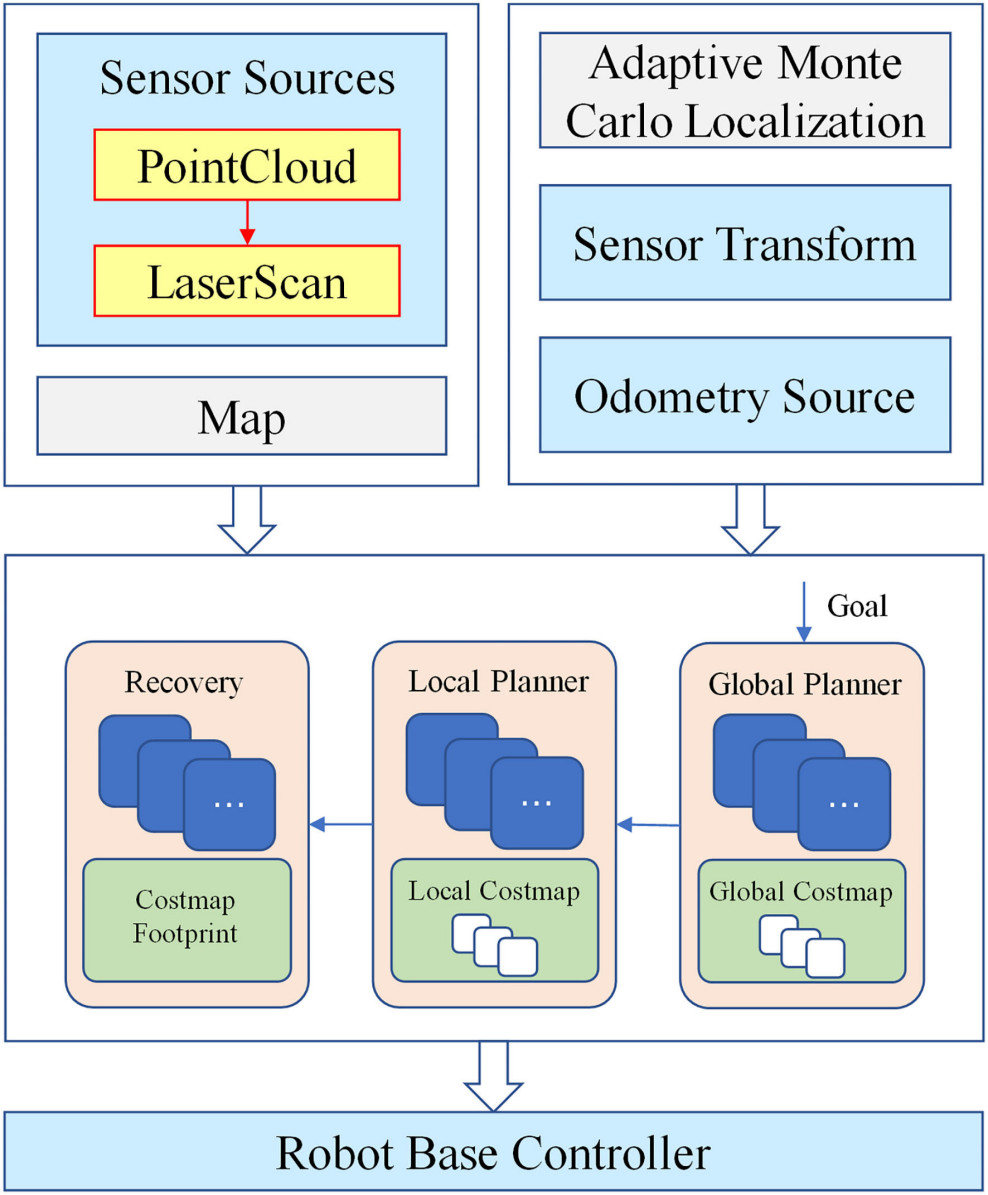
The framework of navigation functions.

The framework of robot navigation function realization in the greenhouse is shown in Figure 3. First, the 3D point cloud data collected by 3D Lidar were filtered and fused into 2D Lidar, and then, the 2D Lidar SLAM algorithm was used to construct the greenhouse environment map based on the data. The positioning of the robot in an unknown environment was mainly realized by the Adaptive Monte Carlo Localization (AMCL) algorithm. Robot target point path planning was the focus of navigation function realization. It was divided into two parts: global path planning and local path planning, which were based on the global cost map and the local cost map, respectively. Finally, the robot integrated the above information in the ROS visualization (RVIZ) tool provided by ROS and used the multi-target navigation settings to realize the robot's mobile navigation in the greenhouse.

#### Multi-Line Lidar Point Cloud Filtering and Fusion

The data transmission between different nodes in ROS is mainly achieved through the communications of Topic, Service, and Parameter Server. The ROS specifies different standard data message types for different sensors, among which the Lidar data is divided into two types: LaserScan.msg (2D Lidar) and PointCloud2.msg (3D Lidar). In this article, both the acceptance and the transmission of Lidar data involved in the robot used the Topic communication based on TCP. Compared with single-line Lidar, multi-line Lidar contains 3D coordinates and intensity of each point cloud data for each frame. By setting the point cloud conversion node, on the one hand, Lidar's topic can be subscribed through the Topic communication and can constantly accept the 3D point cloud data. The 3D point cloud data beyond the height range of the robot's movement height is filtered, while the 3D point cloud data within the height range is fused layer by layer, and the points with the shortest distance within the same height range are selected as the last output data. On the other hand, the point cloud conversion node releases the Laser topic to the 2D Lidar SLAM node and outputs the filtered and fused Lidar data, thus, greatly retaining the key point environment information within the range of the robot movement in the map.

**Figure 4:**
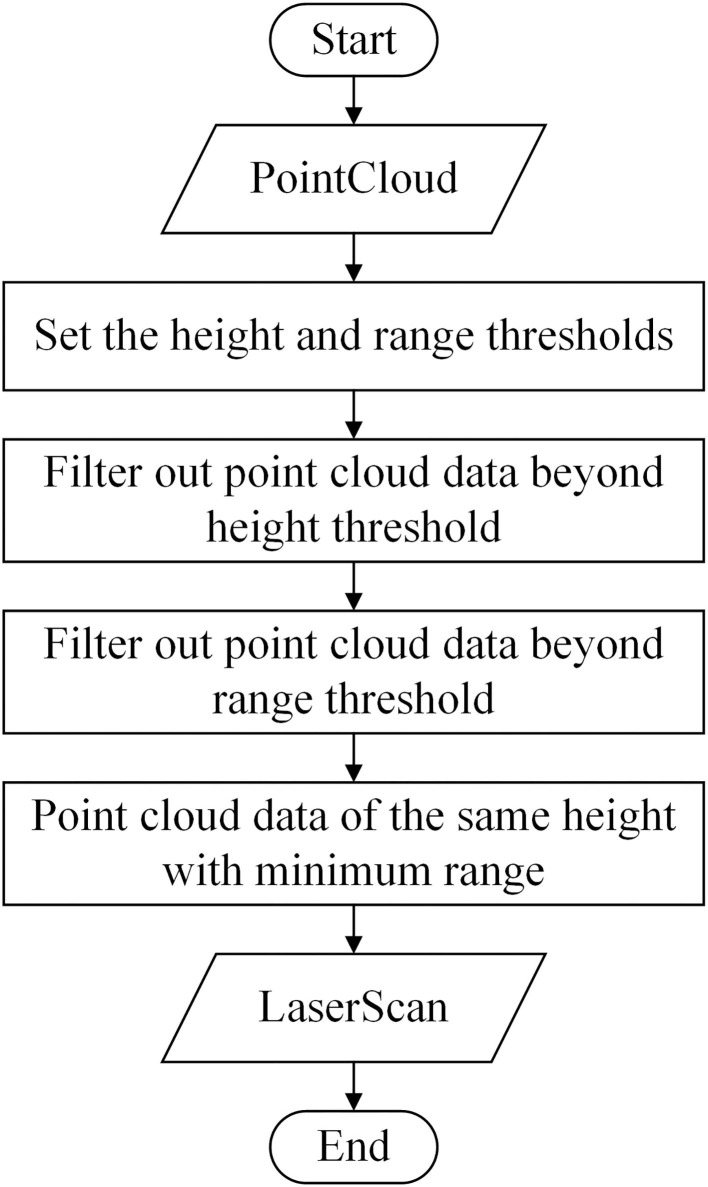
Flowchart of point cloud filtering and fusion.

The specific process of the multi-line Lidar point cloud filtering and fusion algorithm used by the robot is shown in Figure 4. Each frame of Lidar point cloud data is composed of its corresponding three-dimensional coordinates. The position information of the point cloud is clear after knowing its coordinate information. Firstly, the 16 pairs of point cloud data, whose height and range fall beyond the threshold range, were sequentially filtered by setting the height threshold and the range threshold, and the point cloud data within the threshold are retained. Then, the point cloud data of the same height were compared at certain size angles. Finally, the data with the smallest range were saved as the final collected data. Through this algorithm, the robot could quickly and effectively compress the greenhouse 3D environment into 2D, which provided accurate and stable environmental information for subsequent mapping and navigation.

The higher the frequency and the greater the resolution of Lidar, the more environmental information can be obtained at the same time, but the huge amount of data also increases the burden of data processing for the robot. Considering the amount of Lidar data and the data processing capability of the computer, the Lidar frequency was set to 10 Hz, its horizontal resolution was 0.18°, and the number of points per second was 320,000. To reduce the loss in the process of Lidar data transmission, the angle increment of Lasersacn output by the point cloud conversion node took the same value as the horizontal resolution of Lidar; the scanning angle range was from and −3.14 to 3.14, and the scan topic publishing frequency and Lidar point cloud topic publishing frequency were the same to set to 5 Hz. The height of the robot was 0.3 m, and the Lidar was installed at 0.25 m above the robot. Since the converted Lidar data were a LaserScan on the same plane as the Lidar, the height threshold was set from −0.47 to 0.1 m. The measurement range of the Lidar was from 0.15 to 150 m. Considering the actual size of the greenhouse, the range threshold was finally from 0.15 to 50 m.

#### Environment Map Construction

The environment map construction is an important part of the robot navigation and the control system. The quality of map construction directly affects the accuracy of the robot in the navigation and positioning process. The current popular 2D Lidar SLAM algorithms include Hector SLAM, G mapping, Karto SLAM, etc. By comparing the algorithms in the simulation environment, actual environment, and CPU consumption (Santos et al., 2013; Hess et al., 2016), this study finally chose to refer to the Cartographer SLAM algorithm developed by Google. The algorithm adopts the idea of constructing a global map based on sub-maps; each frame of the laser scan data obtained is inserted into the submap at the best-estimated position using a scan match, and the generated submap performs a local loop closure and a global loop by a branch-and-bound approach and several precomputed grids. Cartographer is more advantageous in terms of mapping effects, data processing, and sensor requirements. After the algorithm processing, the robot can finally generate a 2D grid map with a precision of 5 cm.

The Cartographer algorithm is mainly composed of two parts: Local SLAM and Global SLAM. In the part of Local SLAM, odometry and IMU data are used to calculate the estimation value of posture of the robot ξ, ξ = (ξ_*x*_, ξ_*y*_, ξ_ϑ_), and this value is used as the initial value to scan and to match the Lidar data, and the scanned data is recorded as $H={\left\{h_{k}\right\}}_{k=1,\cdots \;,k},h_{k}\in R^{2}$. After the motion filtering, each frame of Lidar data is superimposed to form a submap. The position of {*h*_*k*_} in submap is expressed as *T*_ξ_, and its transformation formula is as follows:


(1) $$\begin{array}{r} T_{\xi }p\;=\;\underset{R_{\xi }}{\underset{︸}{\left(\begin{array}{cc} cos_{\xi_{\theta }} & -sin_{\xi_{\theta }}\\ sin_{\xi_{\theta }} & cos_{\xi_{\theta }} \end{array}\right)}}\;p\;+\;\underset{t_{\xi }}{\underset{︸}{\left(\begin{array}{c} \xi_{x}\\ \xi_{y} \end{array}\right)}} \end{array}$$

where *p* represents the coordinates of the robot before the transform, *R*_ξ_ represents the rotation matrix, and the *t*_ξ_ represents the translation matrix.

The part of Global SLAM is responsible for the loopback detection and back-end optimization, so that small submaps form a whole Global map. The optimization problem of loopback is a nonlinear least squares problem, which can be described as


(2) $$\begin{array}{r} \arg\min_{E^{m},E^{s}}\frac{1}{2}\sum_{ij}\rho \left(E^{2}\left(\xi_{i}^{m},\;\xi_{j}^{s};\Sigma_{ij},\xi_{ij}\right)\right) \end{array}$$

where $E^{m}={\left\{\xi_{i}^{m}\right\}}_{i=1,\cdots \;,m}$ is the submap posture, $E^{s}={\left\{\xi_{j}^{s}\right\}}_{j=1,\cdots \;,n}$ is the scan posture, ρ is the loss function, *E* is the residual function, and these postures are all in the world coordinate system.

To obtain a more accurate map, the robot used the IMU coordinate system as the ROS coordinate system tracked by the SLAM algorithm and the odometer to publish the pose coordinates. The robot controlled the node through a keyboard to walk in the greenhouse at a speed of 0.4 m/s to build a map. After the map was completed, the global environment map was saved in pgm format through the map server node. In the picture, the probability of the existence of obstacles was represented by different grayscale values and for subsequent navigation.

#### Path Planning

The path planning of the robot in the greenhouse is completed based on the built map; however, the original map is static and the obstacle information on the map cannot be updated in real time. Therefore, a costmap is introduced in the robot's path planning. Costmap is mainly composed of Static Map Layer, Obstacle Map Layer, and Inflation Layer. The Static Map Layer usually includes the loaded original map data. The Obstacle Map Layer includes the real-time obstacle information detected by sensors. The Inflation Layer expands the obstacle according to the expansion radius parameter to make the robot move more safely.

The path planning of the robot in the greenhouse was divided into two parts: global path planning and local path planning. The robot first used the global path planner to plan a rough path in combination with the global costmap, then the local path planner divided the planned path into many small paths on this basis, and finally, the local path planner performed the local path planning by referring to the local costmap. In this way, not only the obstacles saved in the map could be avoided in the global path planning, but also the new obstacles and dynamic obstacles could be avoided in the local path planning. The robot navigation target points setting was realized through the Publish Point function in the RVIZ visualization interface. When the mouse was clicked on the RVIZ map interface using the Publish Point function, the Topic communication would be used to publish the location information of the point in the map to the outside world. By setting the node to subscribe to the topic and store the set target points in sequence, the target point information was further published to the navigation node in sequence, and the path planning and multi-target point navigation were completed one by one.

**Figure 5:**
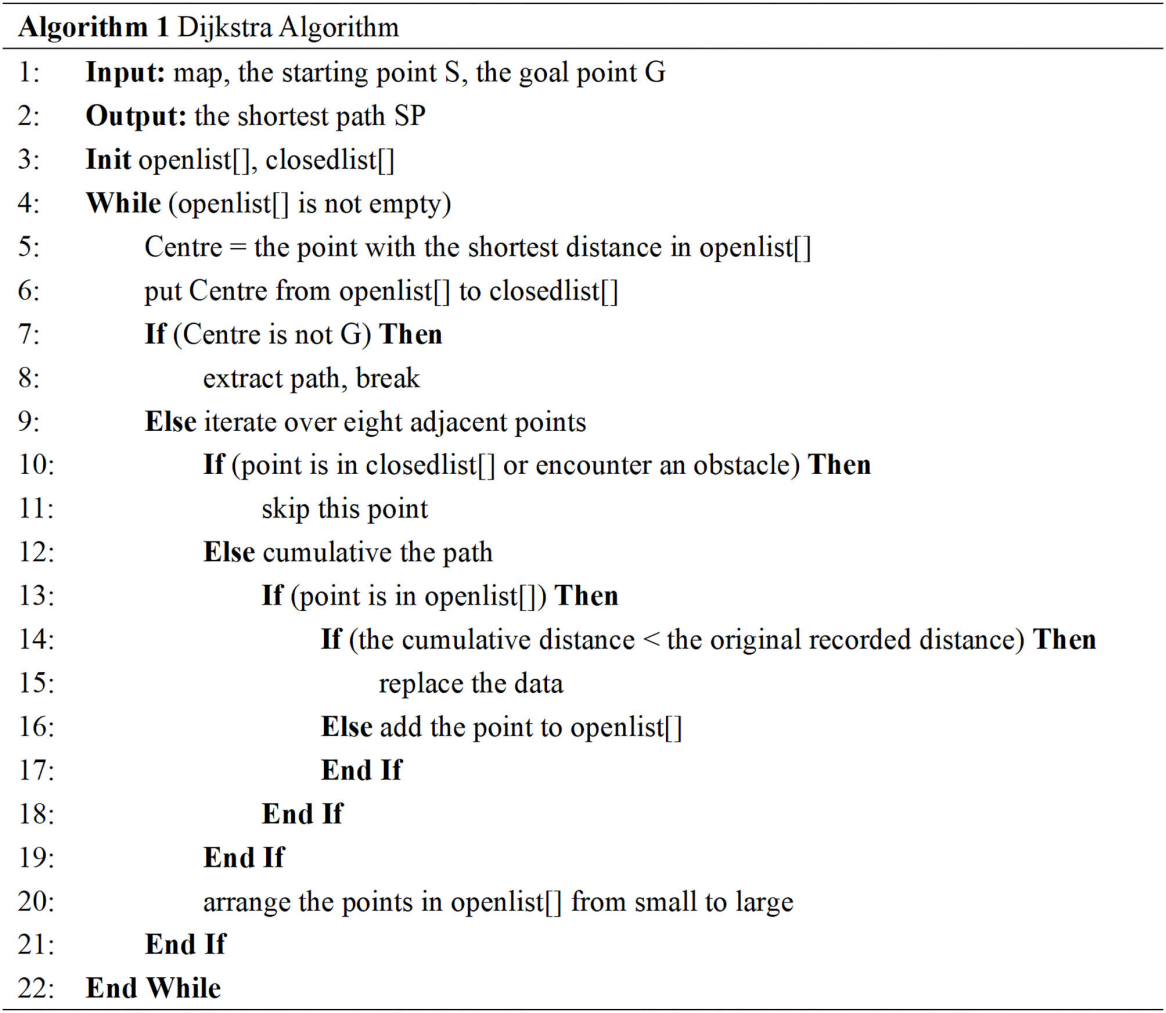
The pseudo-code of the Dijkstra algorithm.

The global path planning of the robot adopts the Dijkstra algorithm, and the algorithm is shown in Figure 5. First, the starting point and the goal point of the robot navigation is set; then, two arrays to store the points of the path to be determined and the points of the determined path are set up, respectively; and next, the distance between the center point and the adjacent 8 points is calculated using the starting point as the center point. Later, we stored the point with the smallest distance, considered the point with the smallest distance as the center point, and calculated the distance between the starting point and the adjacent points from the center point again. For the points that have been calculated, we selected the solution with the smallest distance. In this way, the adjacent points are continuously calculated until the target point is encountered, and the shortest path planning route is output. In general, the algorithm calculates and compares the weights of nodes in the graph from the global perspective to obtain the global shortest path.

**Figure 6:**
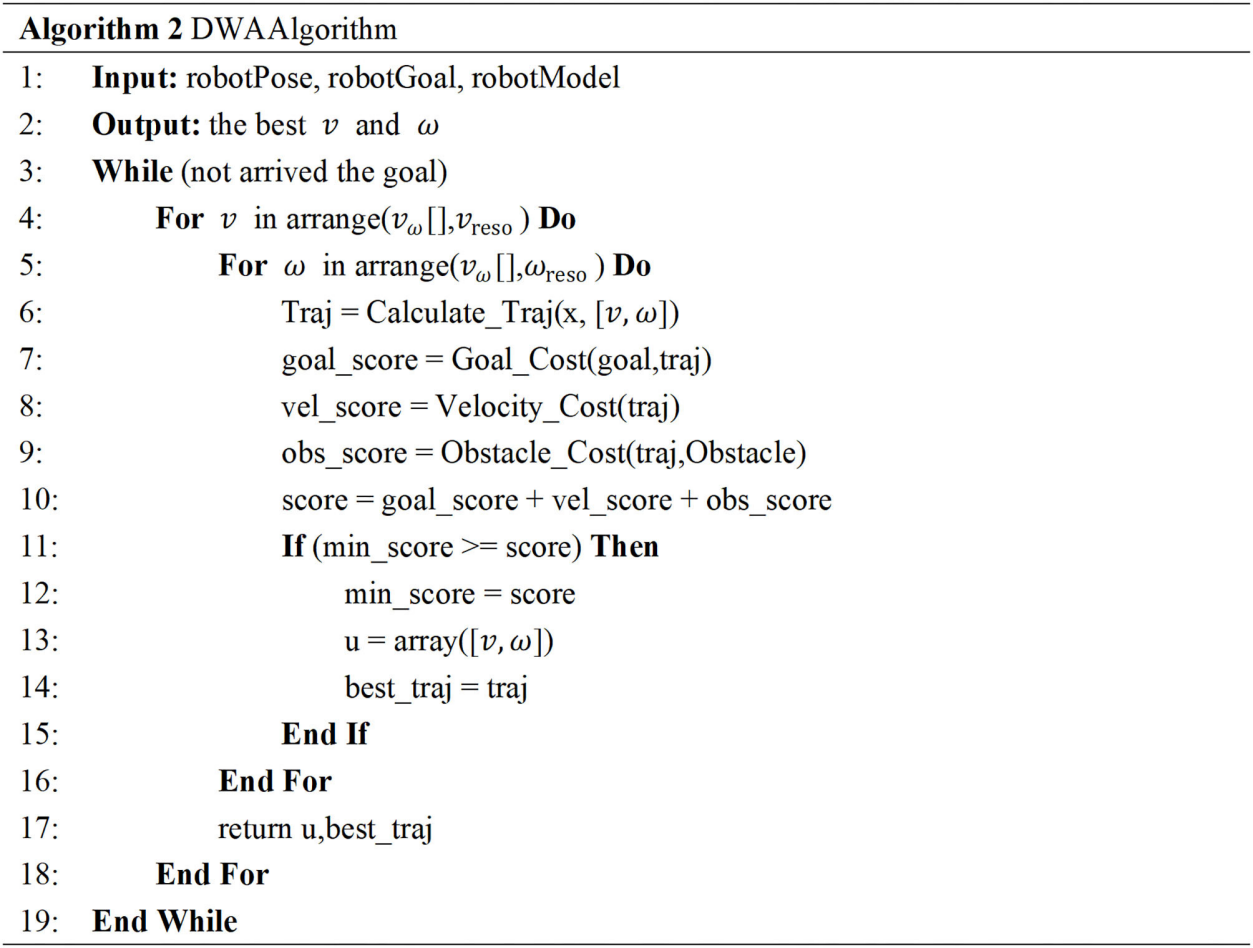
The pseudo-code of the DWA algorithm.

The Dynamic Window Approach (DWA) algorithm is adopted for the robot's local path planning, and the algorithm is shown in Figure 6. The main process includes four parts: initialization, sampling speed samples, sample scoring, and release plan. First, we load the instance of the subclass in BaseLocalPlanner through the class loading module and call its initialization function to obtain the initial state information of the robot and further obtain the trajectory motion model of the robot. Based on the trajectory motion model, the robot can calculate its motion trajectory according to its motion speed. In order to obtain a sample of the robot motion speed, it is necessary to collect the linear speed and the angular speed of each dimension of the robot through sensors within a certain time interval and store them in the corresponding container in the form of a structure. After obtaining the robot speed sample, the corresponding motion trajectory is deduced according to the robot sampling speed simulation, and each trajectory is evaluated through the trajectory evaluation function, as shown in formula (3).


(3) $$\begin{array}{r} G\left(v,\omega \right)\;=\;\max\left(\varphi head\left(v,\omega \right)\;+\;\beta dist\left(v,\omega \right)\;+\;\delta velo\left(v,\omega \right)\right) \end{array}$$

where *head*(*v*, ω) and *velo*(*v*, ω) are given by the formula


(4) $$\begin{array}{rcl} head\left(v,\omega \right) & = & 1\;-\;|\theta |/\pi \end{array}$$

(5) $$\begin{array}{rcl} velo\left(v,\omega \right) & = & v/\pi \end{array}$$

where *head*(*v*, ω) represents the proximity between the velocity trajectory and the target point, and θ represents the included angle between the motion direction and the destination point. The *dist*(*v*, ω) represents the distance from the motion estimation to the nearest obstacle at this sampling speed. If there is no obstacle, the value is a constant. *The velo*(*v*, ω) represents the forward efficiency of the robot under this speed group. The three constant term factors, φ, β, and δ, represent the proportion of the three sub-items in the evaluation function, respectively. Adjusting the three constant factors will affect the actions of the robot in local obstacle avoidance. Finally, all speed groups are evaluated by the above formula, and the speed with the highest score is selected as the current speed command for the movement of the robot.

## Experimental Results and Discussion

**Figure 7:**
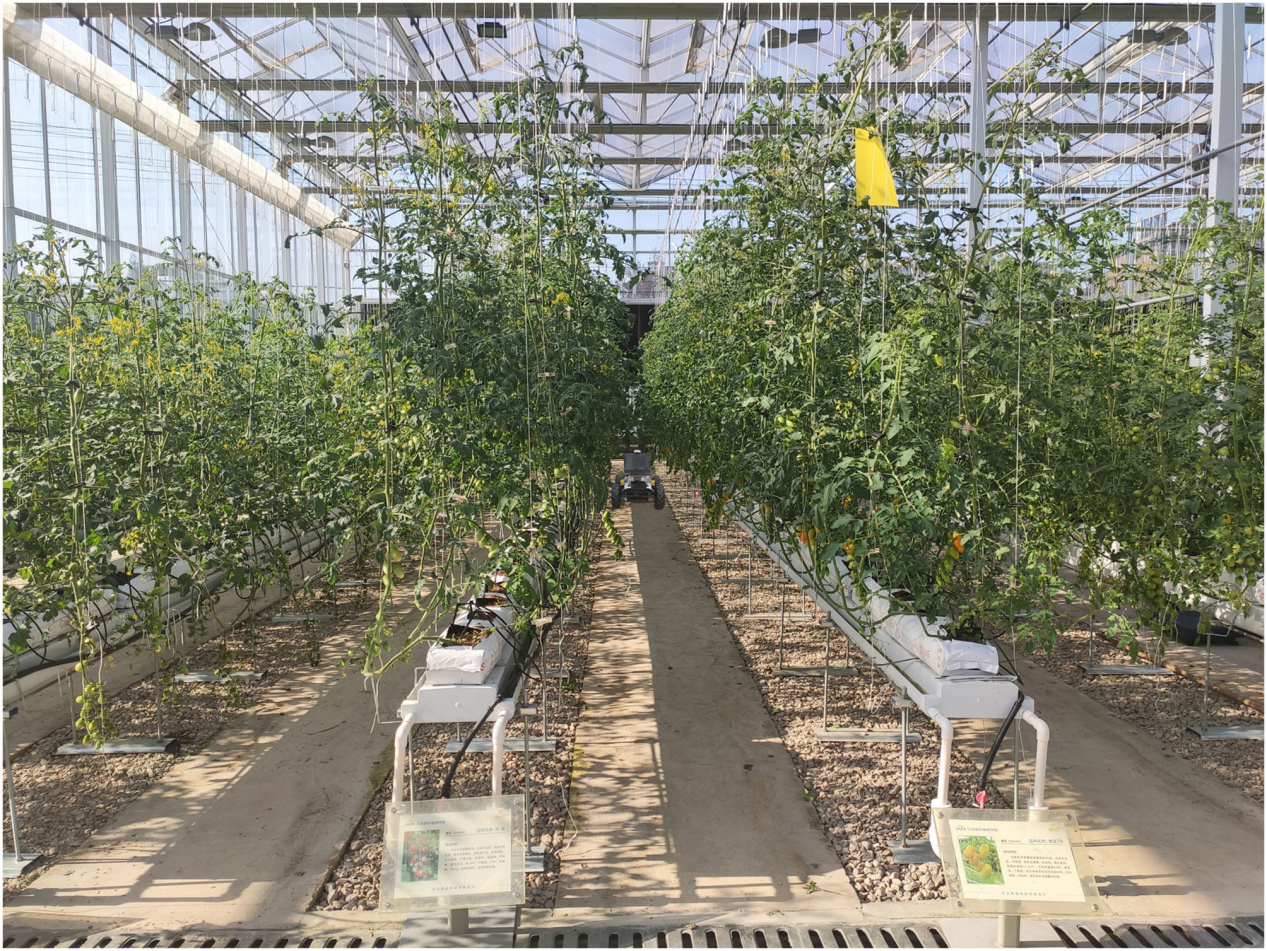
Test environment and robot.

The test site is in the Institute of Agricultural Facilities and Equipment, Jiangsu Academy of Agricultural Sciences, Jiangsu province, China, as shown in Figure 7. The experimental greenhouse is a glass greenhouse, in which tomatoes are grown in the cultivation tanks, and the row spacing between the cultivation tanks is 1 m.

### Robot Positioning Accuracy Test

**Figure 8:**
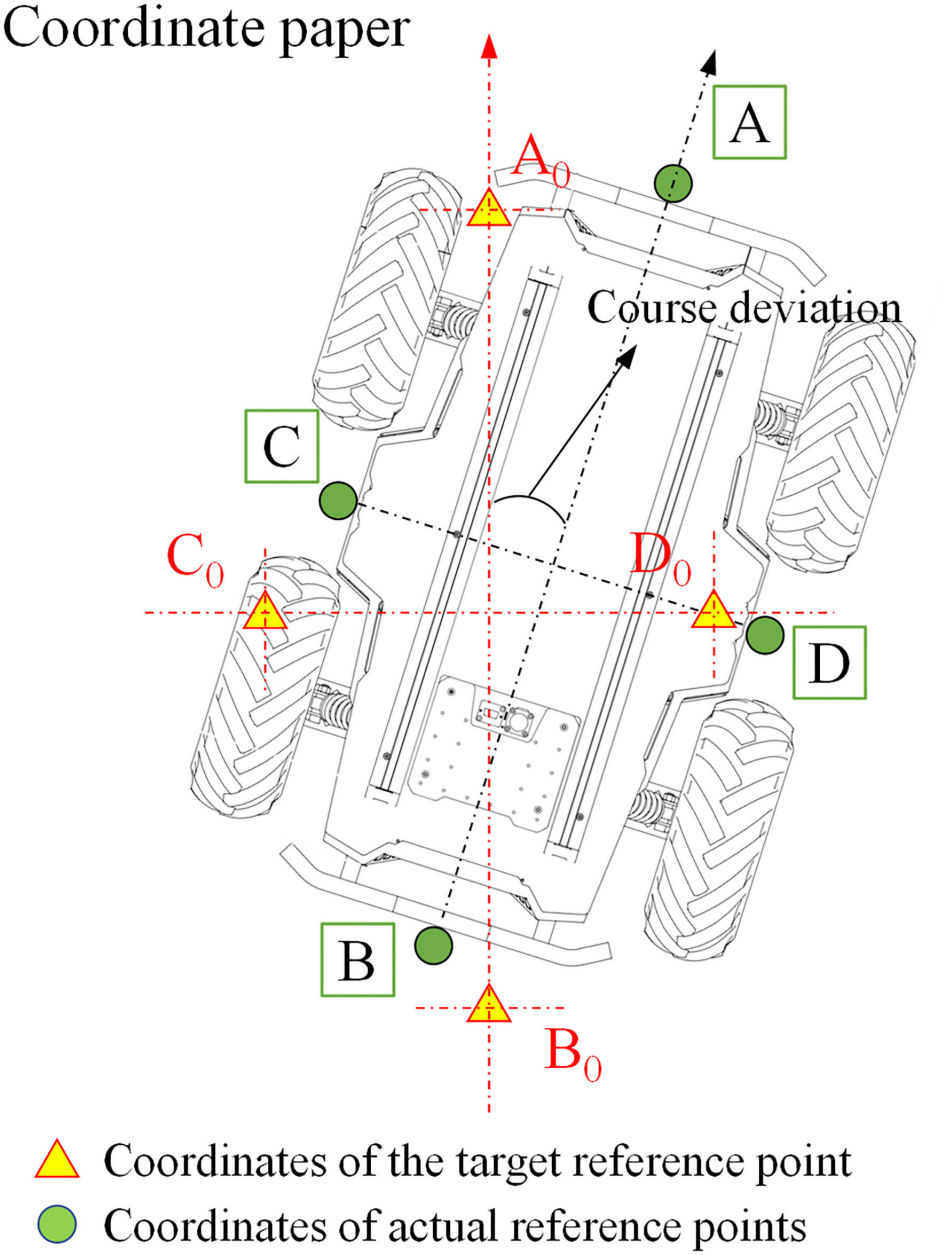
Schematic diagram of the positioning accuracy test.

The robot positioning accuracy test is an effective way to verify the precision and the reliability of the robot navigation system. To accurately measure the position and the posture of the robot at the target points, four target points in the robot greenhouse navigation path were randomly selected, and the positioning coordinate tags were pasted on these four target points. The schematic diagram of the robot positioning accuracy test is shown in Figure 8. The four points, such as the front, rear, left, and right, of the robot were randomly selected as the relative reference positions, and a cross laser (Qy-620, Huimei, Dongguan, China) on each of the four points was installed. After the robot reaches the target point and stops, the coordinate position of the laser shot by the laser was recorded accurately on the coordinate tags. The robot completed 10 complete navigation and positioning tests in sequence at a speed of 0.4 m/s. After each test, the robot needed to be repositioned to its initial position to avoid the accumulation of errors during the test and to ensure the independence of the test.

**Figure 9:**
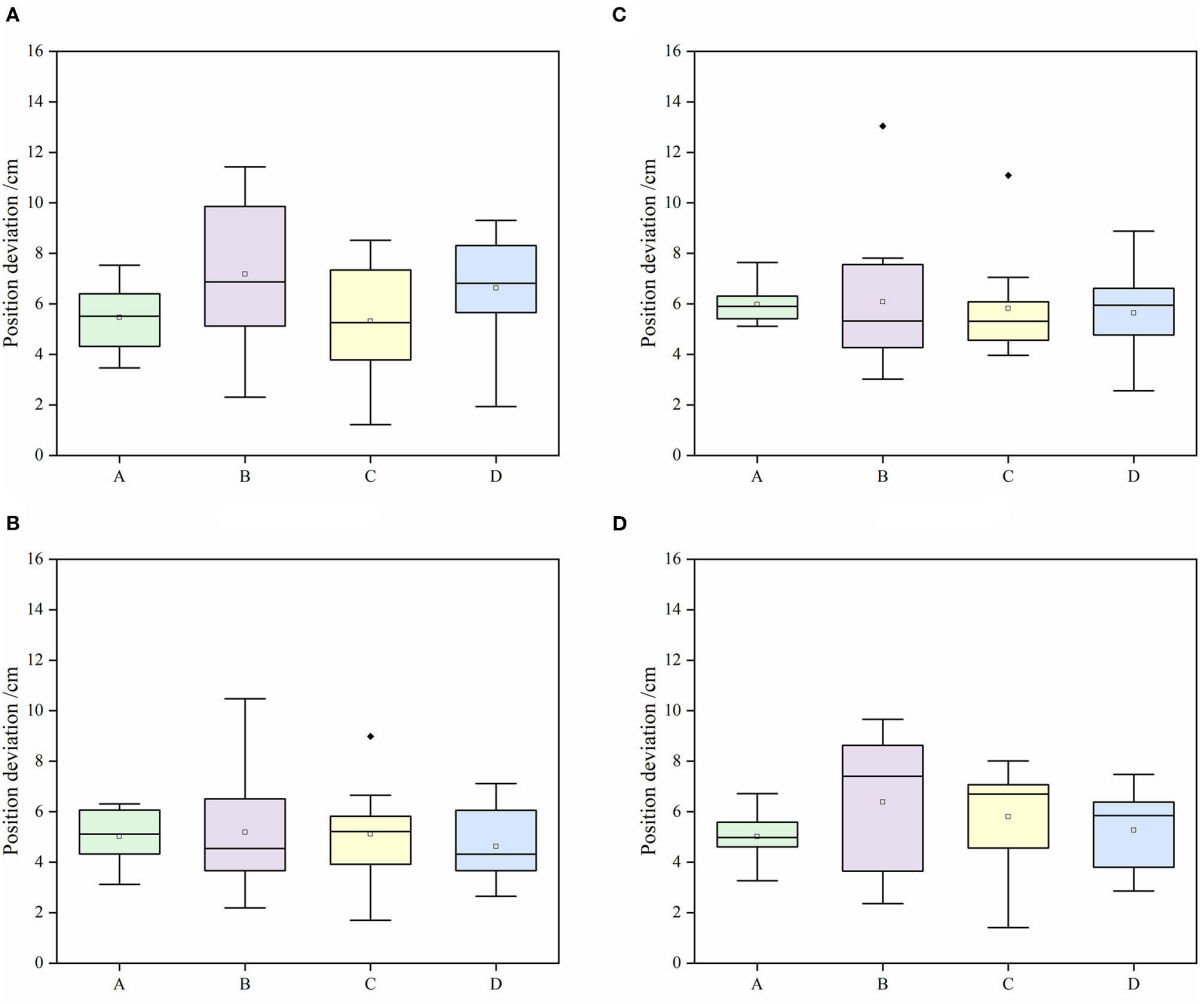
Positioning deviation of the robot at each target point. **(A)** Target 1, **(B)** target 2, **(C)** target 3, **(D)** target 4.

**Figure 10:**
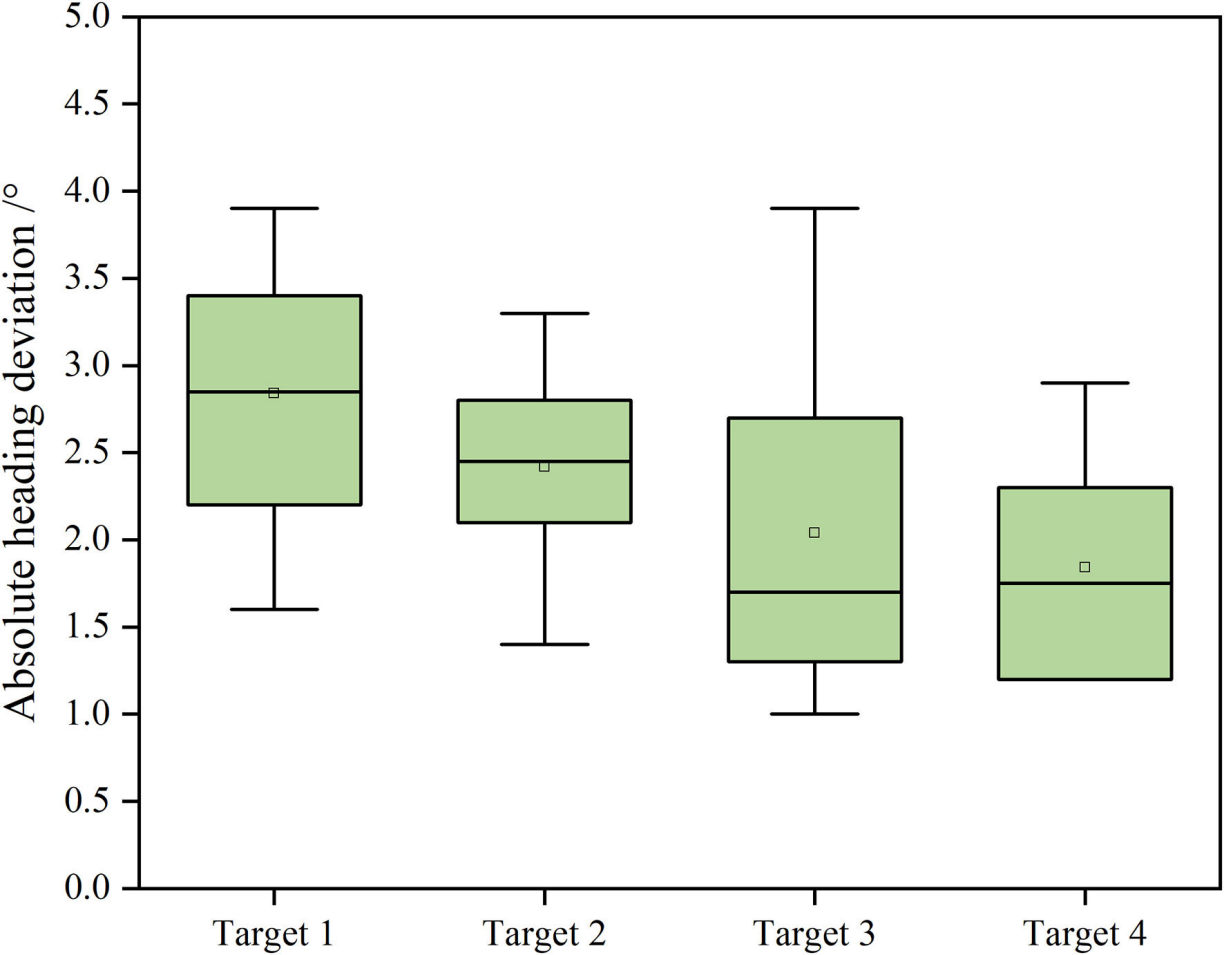
Absolute heading deviation of the robot at each target point.

Quantitative analysis is made on the navigation accuracy of the robot. The position deviation and absolute heading deviation of the four relative reference positions on the robot at the four target points are shown in Figures 9, 10.

It can be seen from Figures 9, 10 that at a speed of 0.4 m/s, the average absolute position deviation of the robot is less than 8 cm, and the SD is less than 3 cm. The average heading deviation of the robot is less than 3°, and the SD is less than 1°. The precision can meet the requirements of the robot positioning in a greenhouse environment. Although the average positioning accuracy of the robot at the four points is not very different, it is still found that target point 2 and point 3 have abnormal points in the test after comparison. By analyzing the position of the target points, the target point 1 and point 4 are close to the two ends of the cultivation tanks, the target point 2 and point 3 are close to the middle of the cultivation tanks, and the structured feature information of target point 1 and point 4 are more than the target point 2 and point 3, such as greenhouse walls and air conditioners, and the environmental information around target point 2 and point 3 are mostly from cultivation tanks and plant leaf walls, with high similarity. So, we think that adding some different objects with structural features in different positions in the greenhouse can improve the positioning accuracy of the robot. The localization system of the robot was implemented based on AMCL, which used particle filters to track the robot's pose against a known map. In general, the more particles there are, the more accurate the positioning is, but the higher the CPU consumption is as well. To achieve a more accurate positioning of the robot under the existing computing power of the robot, we set the maximum number of particles allowed by the positioning algorithm to 4,000 and the minimum number of particles to 1,000. Through continuous testing, the robot had a good performance under this parameter.

### Robot Navigation Accuracy Test

**Figure 11:**
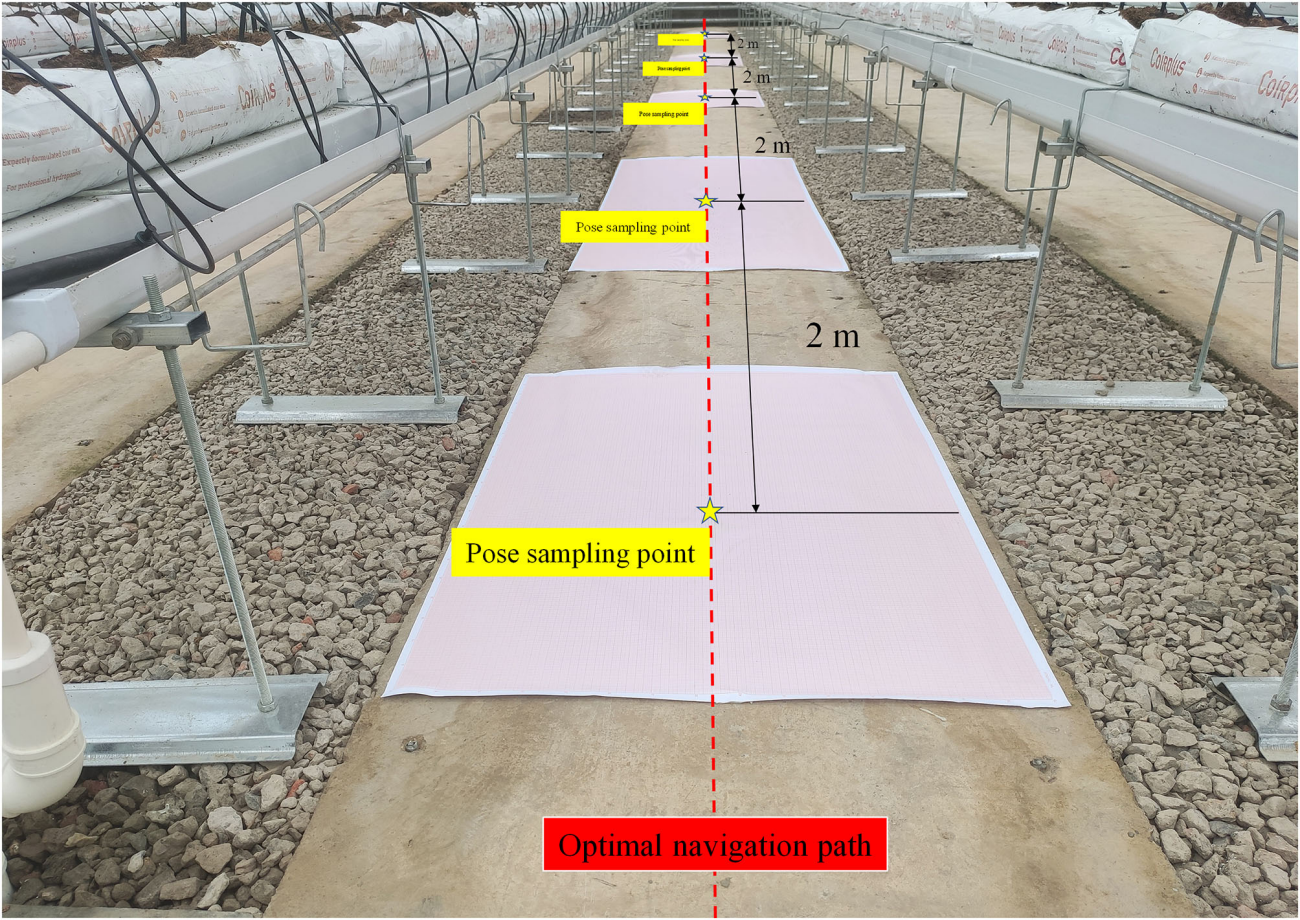
The navigation accuracy test scenario.

The robot navigation accuracy test is the most direct and effective method to test the robot navigation system. The two most important parameters are the lateral deviation and the heading deviation between the robot and the planned path during the movement process. To obtain the lateral deviation and the heading deviation of the robot, initially, obstacles on the road between the greenhouse rows were moved away, and then, the robot navigation target points are set. According to the principle of global path and local path planning algorithms, the optimal navigation path of the robot is the straight line between two target points. Hence, as shown in Figure 11, a posture sampling point was set every 2 m on the planned paths. To accurately collect the position information of the robot, two cross lasers (Qy-620, Huimei, Dongguan, China) were installed in the front and the rear of the longitudinal center line of the robot. When the robot reached each posture sampling point, it stayed there for 5 s in that position for each sampling point, and the positions of the laser on the coordinate tags were recorded accurately. The robot completed the navigation task at the speed of 0.2, 0.4, and 0.6 m/s, respectively. The experiment was repeated three times at each speed.

**Figure 12:**
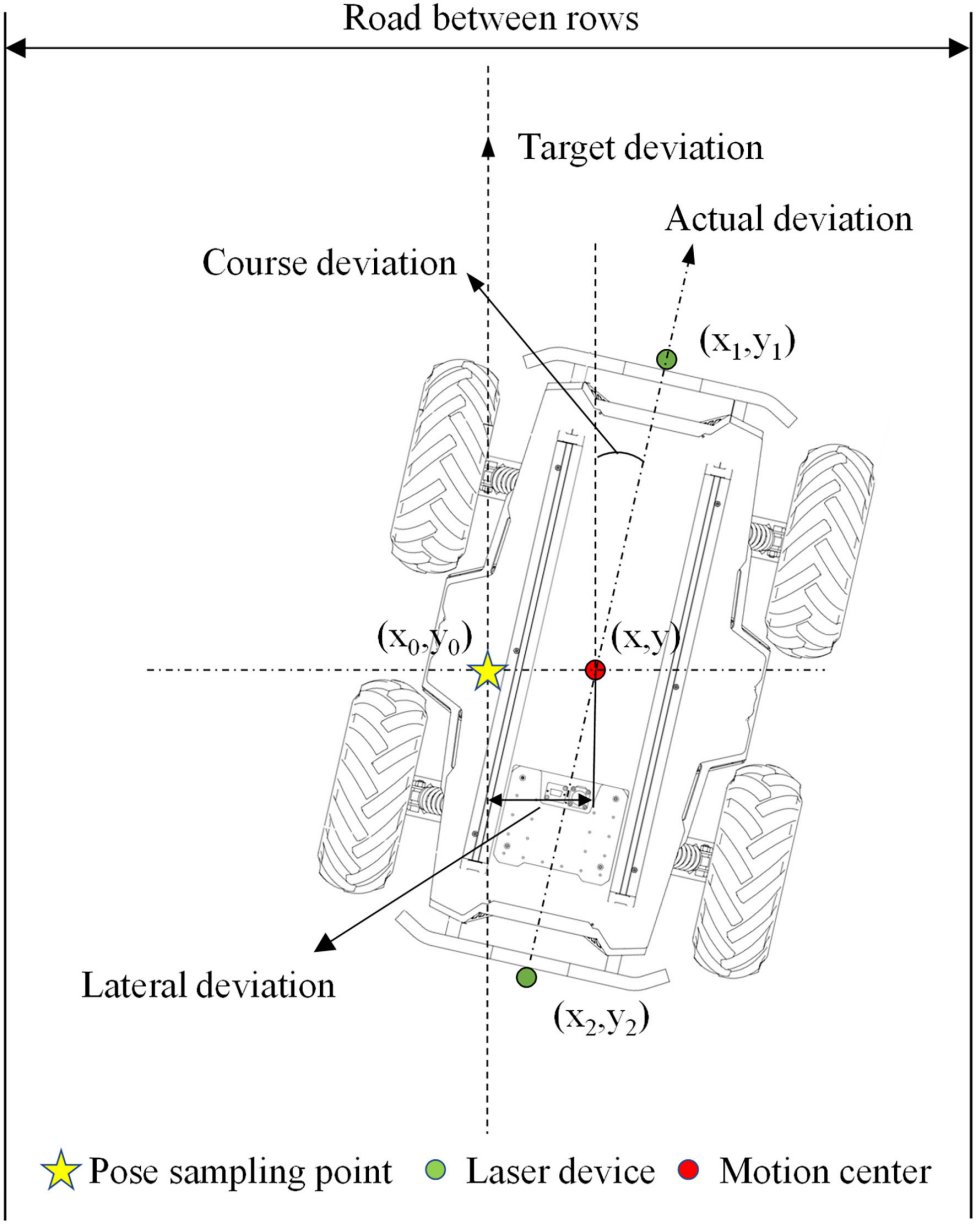
Schematic diagram of the navigation accuracy test.

As shown in Figure 12, the cartesian coordinate system is established on the coordinate paper with the sampling point (*x*_0_, *y*_0_) as the origin, and the target heading of the robot is set to the positive direction of the Y-axis, while the right direction perpendicular to the target heading is the positive direction of the X axis; thus, the coordinate of the sampling point is (0, 0). Suppose the front laser coordinate is (*x*_1_, *y*_1_), the rear laser coordinate is (*x*_2_, *y*_2_), the robot center coordinate is (*x, y*), then the robot center coordinate is $\left(\frac{x_{1}+x_{2}}{2},\frac{y_{1}+y_{2}}{2}\right)$, the lateral deviation is |*x*|, and the heading deviation is $\arccos\left(\frac{y_{1}-y_{2}}{x_{1}-x_{2}}\right)$.

The robot global path planning step size was set to 0.05 m as the grid length in the grid map, and the global path planning frequency was 1 Hz. After numerous tests, when the robot reached the target point, the distance error from the target point in the x-y plane was set to 0.15 m, and the yaw angle error was set to.1 radians, the robot has the best navigation. When these two errors are set smaller, the robot will always hover near the target point. The simulation time of the robot's local path planning was set to 3 s. If the simulation time is too large, it will easily cause the robot to deviate from the global path, especially when the turning radius is large at startup. On the contrary, when the simulation time is too small, it is easy to cause frequent path planning and consume resources, and even oscillation occurs. The step size of the robot's local path planning was set to 0.025 m. After many tests, the three influencing factors in the speed evaluation function were finally set as: φ = 64, β = 24, *and δ* = 0.5, respectively.

It can be seen from Table 1, with the increase in the moving speed of the robot, both the mean value and SD of the lateral deviation of the robot and the mean value and SD of the heading deviation of the robot gradually increase, and the change rate of each deviation when the speed is greater than 0.4 m/s is greater than the transformation rate when the speed is less than 0.4 m/s. At a speed of 0.6 m, the average lateral deviation of the robot is 4.4 cm higher than the average at 0.4 m/s, and the maximum lateral deviation even reaches 16.8 cm. We guess this is related to the part that we set and the parameters related to local path planning. We have not yet found the specific reason for the increase of the deviation, which will be one of the problems that we need to focus on in the next stages. In general, the average lateral deviation of the robot is less than 9.2 cm, and the SD is less than 5.8 cm. The mean course deviation shall not exceed 2.8°, and the SD shall not exceed 1.2°. As is shown, the precision can meet the requirements of navigation precision of robot in a greenhouse environment.

**Table 1:** Robot navigation deviation.

**Speed/(m/s)**	**Lateral deviation/cm**				**Course deviation/ (** **°** **)**			
	**Minimum**	**Maximum**	**Average**	**Standard deviation**	**Minimum**	**Maximum**	**Average**	**Standard deviation**
0.2	0.8	7.1	2.8	1.7	0	3.5	1.5	1.2
0.4	0.6	9.5	4.8	3.0	0.3	3.8	1.7	1.1
0.6	0.8	16.8	9.2	5.8	0.7	4.9	2.8	1.1

At present, the greenhouse is moving from informatization to intelligence. To meet the good application of intelligent equipment in greenhouses, most of the greenhouse floors have undergone a ground leveling treatment. Therefore, the robot positioning and navigation test experiment designed was selected to be carried out in a greenhouse with flat ground. After the positioning accuracy test and the navigation accuracy test, the mobile robot navigation control system designed had a good performance, which had an inseparable relationship with the greenhouse standard planting mode and flat ground. Since the Lidar was fixed on the robot, the Lidar was always level with the ground. At the same time, we used the 3D Lidar information to convert the 2D information and integrate the IMU information, so the slope of the greenhouse floor had no effect on the robot's navigation. To expand the application of the robot in different types of greenhouses, the next step is to test the robot on an uneven ground. When the robot was mapping in the greenhouse, we found that there were often some water pipes and other equipment on the ground, but these obstacles did not affect the movement of the robot. To ignore the influence of these obstacles on the mapping, we chose to filter the point cloud. During the fusion process, filtering was selected for the point cloud below 8 cm from the ground. In addition, for the odometry information required for robot mapping, we took the average value of the four encoders of the robot as the odometer data of the robot, which could effectively reduce the data error caused by the slippage of individual wheels of the robot.

The path planning of the robot was realized based on the costmap after the inflation of the obstacle. To ensure that the robot did not collide with the obstacle, the inflation radius should be larger than the radius of the robot's circumcircle. The robot we designed was 0.8 m long and 0.6 m wide. When the expansion radius of the costmap is larger than the robot's circumscribed circle, the robot will not be able to realize the inter-row path planning. To solve this problem, we set the inflation radius of the costmap to 0.4 m, so that we could ensure that when the robot navigated between rows in the greenhouse, the path planning trajectory was within 0.1 m to the left and right of the center of the row. Even if the robot moved along the inflated obstacles between rows, it would not collide with the cultivation tank. However, this setting method was very dangerous when the robot turns between rows. At the same time, due to the limitation of the row spacing in the greenhouse, the yaw angle of the robot in the row cannot be greater than 53°. To ensure the safety of the robot when turning, we inserted a safety target point at the turning point of the robot's navigation route, divided the robot's navigation plan into multiple parts, and performed a global path planning and a local path planning for each segment to ensure that the robot would not interact when cultivation tanks collide. When the robot got into a local dilemma between the rows, we chose to let the robot terminate the navigation. Although this processing strategy avoided robot collision, it was not intelligent enough. In the future, we will further develop a more intelligent and effective local path processing strategy.

## Conclusion

The proposed autonomous navigation system for the greenhouse mobile robot was designed based on 3D Lidar and 2D Lidar SLAM. The hardware part was mainly composed of 3D Lidar, an IMU, an odometer, and an encoder. The software core control layer was developed based on ROS, and information interaction was realized through a distributed node communication. In order to enhance the safety of the robot during the movement and to reduce the computational power consumption of the computer, 3D environmental information collected by multi-line Lidar was filtered and fused into 2D laser information, and then, localization and map construction were completed using the Cartographer algorithm. After the greenhouse navigation test, the average deviation does not exceed 10 cm, and the average heading deviation does not exceed 3°, which meets the movement requirements of the greenhouse mobile robot. In the process of the robot positioning and navigation, we found that appropriately adding some objects with structured features in the greenhouse environment could effectively improve the positioning accuracy of the robot, and the navigation speed of the robot was closely related to the navigation accuracy. For different navigation speeds, the robot navigation parameters should be reset. At present, this research only solves the simple positioning and navigation of robots in the greenhouse. In the future, we can apply this system to different types of greenhouse mobile robots, and combine the different operating conditions of the robots to develop appropriate navigation strategies based on the existing navigation path planning algorithms. In addition, we can also use 5G, the Cloud Computing Platform, and other modules to further realize the remote control and monitoring of robots.

## Data Availability Statement

The original contributions presented in the study are included in the article/Supplementary Material, further inquiries can be directed to the corresponding author/s.

## Author Contributions

SJ performed most of the experiments with the assistance of SW and ZY. SJ, XL, and MZ designed the study, analyzed the data, and wrote the manuscript. All authors contributed to the study conception and design, read, and approved the final manuscript.

## Funding

This work was supported by the Jiangsu Agricultural Science and Technology Innovation Fund [No. CX(20)1005] and the Jiangsu Modern Agricultural Equipment and Technology Demonstration Promotion Fund (No. NJ2020-23).

## Conflict of Interest

The authors declare that the research was conducted in the absence of any commercial or financial relationships that could be construed as a potential conflict of interest.

## Publisher's Note

All claims expressed in this article are solely those of the authors and do not necessarily represent those of their affiliated organizations, or those of the publisher, the editors and the reviewers. Any product that may be evaluated in this article, or claim that may be made by its manufacturer, is not guaranteed or endorsed by the publisher.
